# Inhibition of the Vancomycin Resistance in *Staphylococcus aureus* in Egypt Using Silver Nanoparticles

**DOI:** 10.1155/2022/7380147

**Published:** 2022-04-30

**Authors:** Nouran M. Salah, Amal E. Saafan, Eman H. Salem, Haddad A. El Rabey, Mohammed A. Alsieni, Fuad A. Alatawi, Adel I. Alalawy, A. B. Abeer Mohammed

**Affiliations:** ^1^Genetic Engineering and Biotechnology Research Institute, University of Sadat City, Egypt; ^2^Department of Microbiology and Immunology, Faculty of Pharmacy, Menoufia University, Shebin Elkom, Menoufia, Egypt; ^3^Department of Microbiology and Immunology, Faculty of Medicine, Menoufia, Egypt; ^4^Department of Biochemistry, Faculty of Science, University of Tabuk, Tabuk, Saudi Arabia; ^5^Pharmacology Department, Faculty of Medicine, King Abdulaziz University, Jeddah, Saudi Arabia University, Shebin Elkom, Egypt; ^6^Department of Biology, Faculty of Science, University of Tabuk, Tabuk, Saudi Arabia

## Abstract

*Staphylococcus aureus* is a major human pathogen that is sometimes resistant to vancomycin. In this study, the prevalence of vancomycin-resistant *Staphylococcus aureus* (VRSA) was studied. 100 isolates of *S*. *aureus* were identified based on biochemical and molecular evidence. The antibiotic susceptibility of the studied isolates was tested against 13 antibiotics by the disc diffusion method that showed 24 vancomycin-resistant isolates. The minimum inhibitory concentrations (MICs) were estimated by the agar dilution method to determine vancomycin intermediate-resistant *S*. *aureus* (VISA) and VRSA. The resistance gene cluster (*vanA*, *vanR*, *vanH*, and *vanY*) was amplified by PCR and then sequenced. Amplification of *vanA* and *vanR* genes showed that they are present in 21.4% and 14.3% of VRSA isolates, respectively, whereas none of the studied genes has been detected in VISA strains. A significant antimicrobial effect toward VRSA isolates using silver nanoparticles (AgNPs) synthesized from *S*. *aureus* and rosemary leaves was recorded. This study confirmed the existence of VRSA strains in Egypt. Furthermore, the use of silver nanoparticles inhibits these vancomycin-resistant *S*. *aureus* strains *in vitro*.

## 1. Introduction

The major human pathogen, *Staphylococcus aureus*, was identified more than 130 years ago as the main cause of suppurative abscess as minor soft tissue infections to life-threatening chronic osteomyelitis, pneumonia, endocarditis, and microorganisms linked to substantial mortality and morbidity [1].

The evolution and use of antibiotics such as methicillin and penicillin in the mid-20^th^ century at first worked effectively against *S*. *aureus*. Unfortunately, *S*. *aureus* has quickly acquired resistance to these antibiotics. Methicillin-resistant *S*. *aureus* (MRSA) and penicillin-resistant *S*. *aureus* (PRSA) infections were hard to be treated [2]. By the late 1990s, the epidemic strains of methicillin-resistant *Staphylococcus aureus* (MRSA) that were multidrug resistant had become the most common infectious agents of *S*. *aureus* disease in communities and even in hospitals, and still, glycopeptide antibiotics, such as vancomycin, were the antibacterial agents used for the treatment of MRSA infections [2]. Vancomycin is commonly used in hospitals for treating serious infections caused by MRSA strains by binding to the lipid II dipeptide D-Ala4-D-Ala5 and inhibiting PBP2- and PBP2a-catalyzed transglycosylation and transpeptidation, as well as peptidoglycan remodeling [3].

Since 1980, vancomycin has been used extensively in the United States and other countries, which reflects the rising incidence of MRSA infections in hospitals and explains the concentration on MRSA strains worldwide [4]. In this period of time, MRSA was increased worldwide, and consequently, the use of vancomycin was also increased because of the infections with vancomycin-resistant *S*. *aureus* (VRSA). In 1977, *S*. *aureus* with reduced susceptibility to vancomycin was reported in Japan, followed by the discovery of vancomycin-resistant *S*. *aureus* (VRSA) that was more important because these isolates demonstrated complete vancomycin resistance [2].

The resistance mechanisms of staphylococci may be due to the thickened cell wall, in which many vancomycin molecules were trapped within the cell wall in which the trapped molecules close the peptidoglycan network and finally form a physical barrier to further incoming vancomycin molecules, or identical to the mechanism seen in vancomycin-resistant enterococci (VRE) in which the VRE harbors the vanA operon which encodes for several proteins involved in the synthesis of D-Ala-D-Lac depsipeptide peptidoglycan precursors, which has a low affinity to glycopeptides [5].

Silver nanoparticles (AgNPs) are characterized by bacteriostatic and bactericidal activities. Microbes are extremely effective in the creation of AgNPs, despite the fact that numerous chemical and biochemical approaches are being investigated. Many traditional and upcoming technologies are predicted to benefit from new enzymatic techniques that use bacteria, plants, and fungi to synthesize nanoparticles both intracellularly and extracellularly [6].

The aim of this study was to assess the prevalence of mutations in putative candidate genes for VISA and VRSA and to develop an extracellular biosynthesis of AgNPs using *S*. *aureus* and rosemary leaves. The evaluation of their antimicrobial activity against MRSA and VRSA isolates was also studied.

## 2. Materials and Methods

### 2.1. Isolation of Bacteria

One hundred *S*. *aureus* isolates were obtained from 100 patients from different hospitals in Menoufia during the period from September 2020 to July 2021. Phenotypic and biochemical assays such as gram staining and growth on mannitol salt agar, catalase, coagulase, and DNase tests were used to identify bacterial isolates as *S*. *aureus*. The morphology of the isolates was determined using a light and electron microscope. They were confirmed as *S*. *aureus* by molecular identification PCR using *nuc* gene primers, “confirmatory gene for *S*. *aureus*” [7, 8].

### 2.2. Antibiotic Susceptibility and Determination of MIC

The disc diffusion method was applied on Mueller-Hinton agar (Pronadisa, Spain) for the determination of antibiotic susceptibility based on Clinical and Laboratory Standards Institute (CLSI) guidelines [9]. The antibiotics used for disc diffusion assays are vancomycin (VAN), linezolid (LZD), oxacillin (OX), cefoxitin (FOX), penicillin (PG), azithromycin (AZM), cefpodoxime (CPD), ceftriaxone (CRO), clarithromycin (CLR), ciprofloxacin (CIP), clindamycin (CLI), doxycycline (DOX), erythromycin (ERY), gentamicin (GEN), levofloxacin (LVX), and teicoplanin (TEC). Minimum inhibitory concentrations (MICs) of vancomycin were determined by the agar dilution method on Mueller-Hinton agar. MIC breakpoints for vancomycin were defined according to CLSI guidelines as follows: susceptible, ≤2 *μ*g/ml; intermediate, 4–8 *μ*g/ml; and resistant, ≥16 *μ*g/ml for vancomycin, as well as susceptible, ≤4 *μ*g/ml and resistant, ≥8 *μ*g/ml for vancomycin [9].

### 2.3. Genomic DNA Extraction and PCR Amplification

Genomic DNA was extracted from the vancomycin-resistant clinical isolates using the Thermo Scientific GeneJET DNA Extraction Kit according to the instruction of the suppliers. After extraction, the purity of DNA was assessed using a spectrophotometer. The first PCR run was done using *nuc* gene primers TN1 (F.5′-GACTATTATTGGTTGATCCACCTG-3′) and TN2 (R.5′-GCCTTGACGAACTAAAGCTTCG-3′) with an expected product size of 218 bp. The PCR was performed in a total volume of 25 *μ*l. The PCR amplification was performed as follows: initial denaturation step at 94°C for 4 min and then 35 cycles at 94°C for 1 min, 54°C for 1 min, and 72°C for 1 min. At the end, the PCR product was held at 72°C for 7 min. Furthermore, *S*. *aureus* ATCC 25923 was used as the positive control in this experiment [7].

The second PCR was multiplex PCR using *vanA* cluster genes using the specific v*anA* primers as shown in Table 1. The PCR condition using vancomycin resistance primers was an initial denaturation step at 94°C for 3 min followed by 40 cycles of DNA denaturation at 94°C for 30 sec, with annealing temperature for each pair of primers (as shown in Table 1) for 2 min, and DNA extension at 72°C for 2 min. After that, the PCR product was held at 72°C for 6 min and then stored at 4°C [10].

### 2.4. PCR Product Sequencing

The Genomic DNA Purification Kit (Thermo Scientific, USA) was used to purify DNA, and then a gel extraction kit (Qiagen, Germany) was used to purify the gel. After that, the nanodrop spectrophotometer from Thermo Scientific was used. The large dye terminator cycle sequencing kit (Thermo Scientific, USA) was used to sequence the cycles. Following that, the sequencing reaction was purified using a large dye XTerminator purification kit (Thermo Scientific, USA). Finally, a 3500 Genetic Analyzer for fragment analysis was used to perform Sanger sequencing (Thermo Scientific, USA).

### 2.5. Synthesis of Silver Nanoparticles from *S*. *aureus* and *Rosmarinus officinalis*

*S*. *aureus* strains were inoculated in Mueller-Hinton broth and incubated at 37°C for 24 hours. The culture was centrifuged at 12,000 rpm for 5 minutes, and the supernatant was utilized for the synthesis of AgNPs. For the synthesis of AgNPs, the supernatant was dissolved in sterile distilled water and then transferred to a reaction vessel containing silver nitrate (AgNO_3_) at a concentration of 10^−3^ (1% *v*/*v*). The reaction between this supernatant and Ag^+^ ions was carried out in light circumstances for 5 minutes [11]. For the synthesis of AgNPs from the leaves of *Rosmarinus officinalis*, 15 g of fresh leaves was boiled in 200 ml distilled water for 10 min, and then 100 ml of the extract was added to 0.017 g of silver nitrate with magnetic stirring for 10 min to obtain AgNPs [6, 12].

### 2.6. Characterization of Silver Nanoparticles

#### 2.6.1. Ultraviolet-Visible Spectroscopy

The biological reduction of Ag^+^ ions was monitored using 2 ml aliquots of the reaction mixture, and the UV-vis spectrum of the mixture was measured. UV-vis spectra of the samples were recorded from 200 to 600 nm at room temperature using the UVS-85 spectrophotometer [13].

#### 2.6.2. Transmission Electron Microscopy (TEM)

A transmission electron microscope was used for the characterization of the size, shape, morphology, assembly, and purity of synthesized AgNPs. Samples were prepared for TEM by placing 2-5 *μ*l drops of the sample onto a sheet of parafilm. After that, EM grids were made directly on the sample. The sample drop was wicked away using filter paper, and then the grid was placed on a drop of filtered 2% phosphotungstic acid (PTA) stain at pH 7 and left for 1 min. Finally, the stain was wicked away using filter paper, and grids were placed in a sample petri dish.

#### 2.6.3. Dynamic Light Scattering (DLS)

Dynamic light scattering (DLS) was used to calculate the size and size distribution of particles dispersed in a liquid sample of silver nanoparticles using the PSS-NICOMP 380-ZLS particle sizing system. The samples were diluted 10 times in deionized water before being measured. 250 *μ*l of the suspension was transferred to a disposable low-volume cuvette. Equilibration was done at 25°C for 2 min before measurement [14].

### 2.7. The Antibacterial Effect of Silver Nanoparticles

The antibacterial activity of AgNPs made from *S*. *aureus* and rosemary leaves was investigated using the well diffusion method against vancomycin-resistant *S*. *aureus* strains. On Mueller-Hinton agar plates, gel puncture was employed to form 6 mm diameter wells. Each strain was swabbed uniformly onto individual plates using sterile cotton swabs. Using a micropipette, 20 *μ*l of the nanoparticle solution sample was pipetted into each of the four wells on the plates (similar steps were done for AgNPs synthesized from rosemary in separate plates). To compare the inhibition zones with and without AgNPs, a vancomycin disc was put on the plate before and after immersion in the AgNP solution. The inhibition zones were observed after a 24-hour incubation period at 37°C [6].

### 2.8. Statistical Analysis

Data obtained from the characterization and antimicrobial activity of silver nanoparticles were statistically analyzed using SPSS statistical package version 26 released in 2018 (IBM SPSS Statistics for Windows, IBM Corp.). The data are represented as the mean ± SE. In addition, the paired *t*-test was used to analyze the antimicrobial activity and stability of silver nanoparticles.

## 3. Results

### 3.1. Isolation and Identification of Isolates

**Figure 1:**
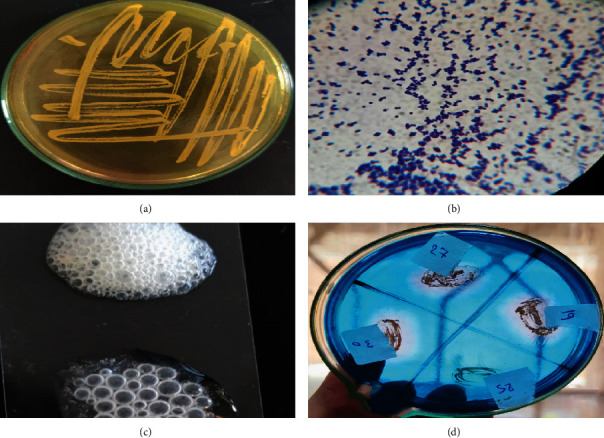
Phenotypic methods for identification of the studied *S*. *aureus*. (a) Growth on mannitol salt agar showed golden-yellow colonies. (b) Gram stain showed purple cells arranged in grape-like clusters. (c) Catalase test resulted in effervescence after the addition of hydrogen peroxide to the fresh growth of staphylococci. (d) DNase test using the toluidine blue indicator resulted in pink hallows around the colonies and a negative result for the control (*S*. *epidermidis*).

**Figure 2:**
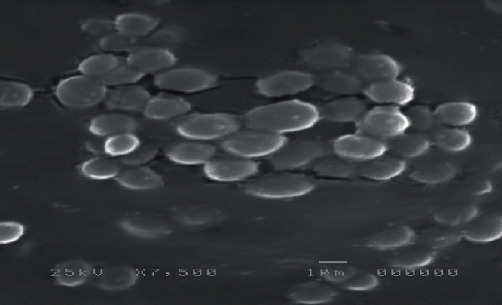
*S*. *aureus* isolate identification using an electron microscope. The cells are round and grouped in clusters like grapes.

In this study, one hundred *Staphylococcus aureus* isolates were obtained from 100 patients (56 males and 44 females) during the period from September 2020 to July 2021. Their ages ranged from six months to seventy years old. They were isolated from different sites: blood (50%), wound swabs (21%), sputum (15%), urine (7%), and pus (7%), as well as from different departments: ICU (27%), internal medicine (26%), PICU (20%), oncology (10%), pediatrics (9%), cardiology (5%), hepatic (2%), and dialysis (1%). All studied isolates were phenotypically identified as *S*. *aureus* as shown in Figures 1 and 2.

### 3.2. Patterns of Resistance

MICs for vancomycin were performed on *S*. *aureus* isolates. Twenty-four isolates were resistant to vancomycin according to MIC by the agar dilution method; out of them, 14 isolates withMIC ≥ 16 *μ*g/ml were for VRSA and 10 isolates with 4-8 *μ*g/ml were for VISA.

### 3.3. PCR Products

**Figure 3:**
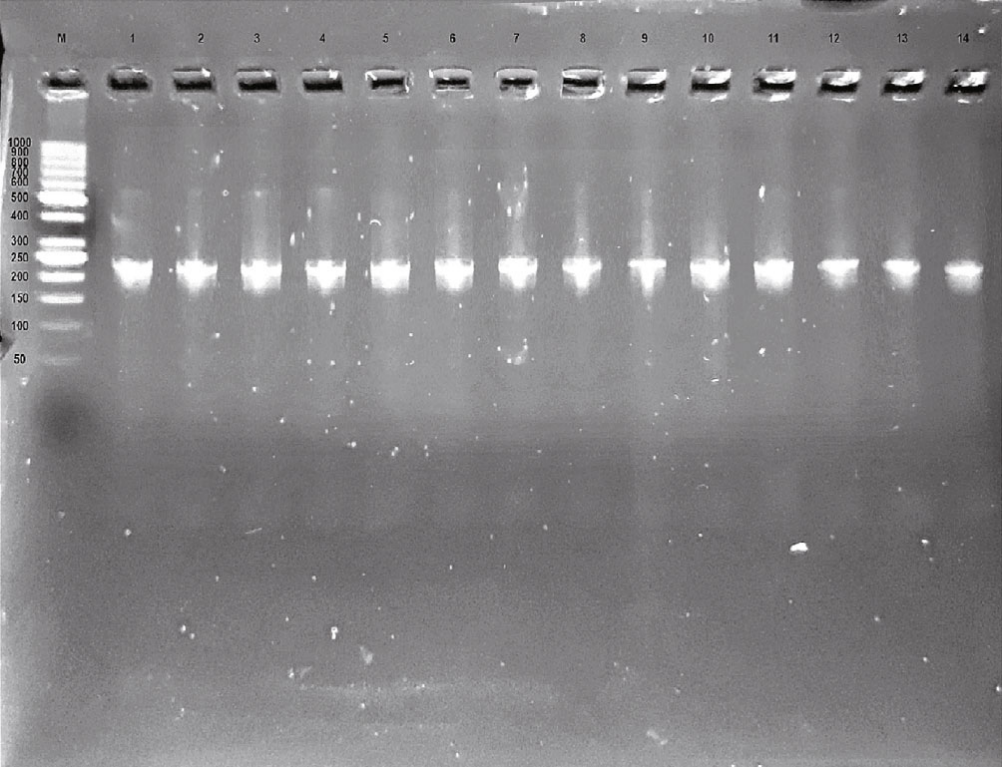
Gel electrophoresis for PCR using a pair of primers for the nuc gene with a product molecular size of 218 bp. All isolates were positive for the *nuc* gene. *S*. *aureus* ATCC 25923 was used as the positive control. Lane M: DNA ladder (50–1000 bp). The figure shows in all lanes the +ve *nuc* gene at 218 bp (lane 1 for the positive control).

**Figure 4:**
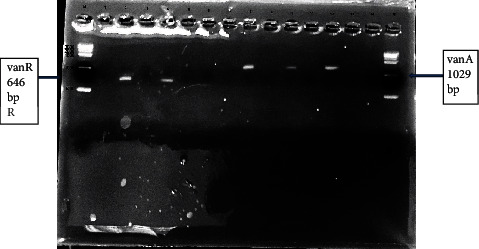
Gel electrophoresis of multiplex PCR amplification products for *vanA* and *vanR* genes. Lane M: 1 kb DNA ladder. The figure shows in lanes 2 and 4 the +ve *vanR* at 646 bp and lanes 8, 10, and 12 the +ve *vanA* at 1029 bp. Lanes 1, 3, 5, 6, 7, 8, 9, 10, 11, 12, 13, and 14 (–ve *vanR*) and lanes 1, 2, 3, 4, 5, 6, 7, 9, 11, 13, and 14 (–ve *vanA*).

As shown in Figure 3, the PCR technique using the *nuc* gene (a confirmatory gene for *S*. *aureus*) was used to confirm that all vancomycin-resistant isolates were *S*. *aureus*. In Figure 4, a multiplex PCR was done to detect the presence of resistant genes for vancomycin in *S*. *aureus* isolates. Only *vanA* and *vanR* genes were detected in our isolates, while *vanH* and *vanY* were not detected in any isolate.

The resulted PCR product of the *vanA* gene was sequenced. The *vanA* gene sequence was 1029 bp. The sequence data of *vanA* was compared with other results using BLASTn search (https://blast.ncbi.nlm.nih.gov/Blast.cgi) that showed >92% similarity to the *vanA* genes isolated from *Staphylococcus* spp. and enterococci. On the other hand, the *vanR* gene was 646 bp in length that showed high similarity (92.03%) to *S*. *aureus* strain 3020.C01 (Acc. No. CP025495.1).

### 3.4. The DLS Spectrum for Silver Nanoparticles

**Figure 5:**
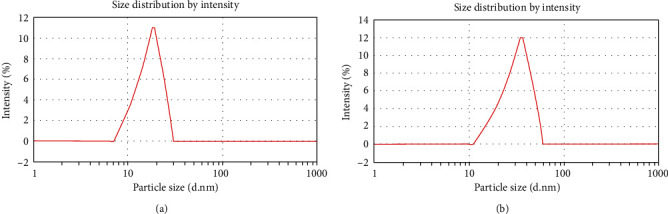
(a) DLS spectrum for silver nanoparticles synthesized using *S*. *aureus*. Average particle size was 19.21 ± 1.00 nm. (b) DLS spectrum for silver nanoparticles synthesized using rosemary leaves. Average particle size was 35.5 ± 2.02 nm.

The DLS spectrum for silver nanoparticles synthesized using *S*. *aureus* gave an average particle size of 19.21 ± 1.00 nm as shown in Figure 5(a), whereas the DLS spectrum for silver nanoparticles synthesized using rosemary leaves gave an average particle size of 35.5 ± 2.02 nm as shown in Figure 5(b).

### 3.5. Antimicrobial Effect of Silver Nanoparticles (AgNPs)

**Figure 6:**
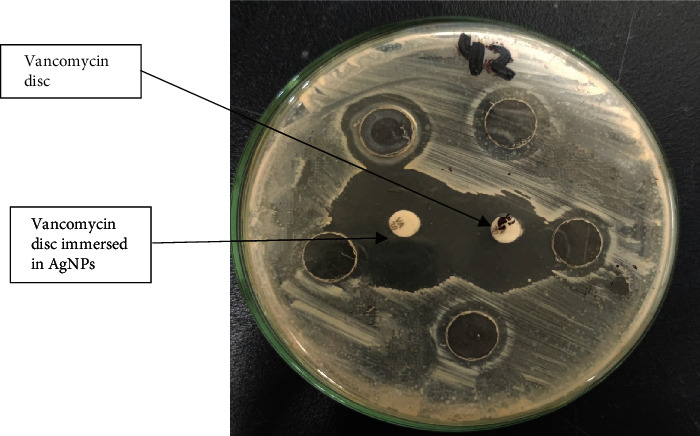
Antimicrobial effect of AgNPs synthesized from *S*. *aureus* against LRSA strain.

**Figure 7:**
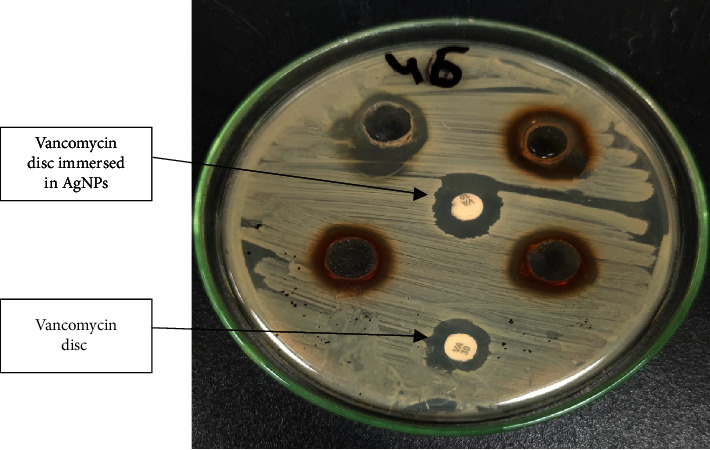
Antimicrobial effect of AgNPs synthesized from *rosemary leaves* and the difference in inhibition zones of vancomycin discs alone and vancomycin discs immersed in AgNPs.

The emergence of a yellowish-brown color in the silver nitrate solution following the addition of the *S*. *aureus* supernatant indicates silver nanoparticle synthesis, whereas the solution stays colorless without *S*. *aureus* supernatant solution as shown in the supplementary file Figure 1. When administered to VRSA using the well diffusion method, silver nanoparticles produced using *S*. *aureus* and rosemary leaves demonstrated a significant antimicrobial activity as shown in Figures 6 and 7. The efficacy of silver nanoparticles synthesized from both *S*. *aureus* and rosemary leaves toward vancomycin-resistant S. *aureus* strains using the well diffusion method was tested after the synthesis of nanoparticles for 24 hours, 1 month, 3 months, and 6 months. The diameters of inhibition zones were nearly similar with no statistically significant difference in the effect. This indicates that silver nanoparticles remain stable. Tables 2(a) and 2(b) show the antimicrobial activity of AgNPs synthesized using *S*. *aureus* and rosemary leaves, respectively. The mean zones of inhibition in both AgNPs of *S*. *aureus* and rosemary indicate that there is an antimicrobial effect of AgNPs. The paired *t*-test was used for comparing zones of inhibition. There was a significant (*P* value < 0.05) increase in the mean zones of inhibition of AgNPs compared with the zones of inhibition of vancomycin discs in both AgNPs synthesized from *S*. *aureus* and rosemary leaves.

In addition, the zones of inhibition of vancomycin discs immersed in AgNPs were increased compared to the zones of inhibition of vancomycin discs alone with statistical significance (*P* value < 0.05) in both AgNPs synthesized using *S*. *aureus* and rosemary leaves. Mean inhibition zones obtained from AgNPs synthesized using *S*. *aureus* were less than mean inhibition zones obtained from AgNPs synthesized using rosemary leaves with a statistically significant result (*P* value = 0.008, SEM = 0.750) which indicates that the AgNPs synthesized using rosemary leaves were more effective than those synthesized using *S*. *aureus*.

On the other hand, the mean values and standard errors for all zones of inhibition for AgNPs and vancomycin are shown in Table 3. The highest zone of inhibition was shown in vancomycin+AgNPs from rosemary leaves, whereas the lowest one was shown in vancomycin.

The efficacy of silver nanoparticles synthesized from both *S*. *aureus* and rosemary leaves toward vancomycin-resistant *S*. *aureus* strains using the well diffusion method was tested after 24 hours, 1 month, 3 months, and 6 months from the synthesis of the nanoparticles as shown in Tables 4(a) and 4(b). The diameter of inhibition zones remained nearly similar with no statistically significant difference in their antimicrobial efficacy. This indicates that silver nanoparticles remained stable.

**Figure 8:**
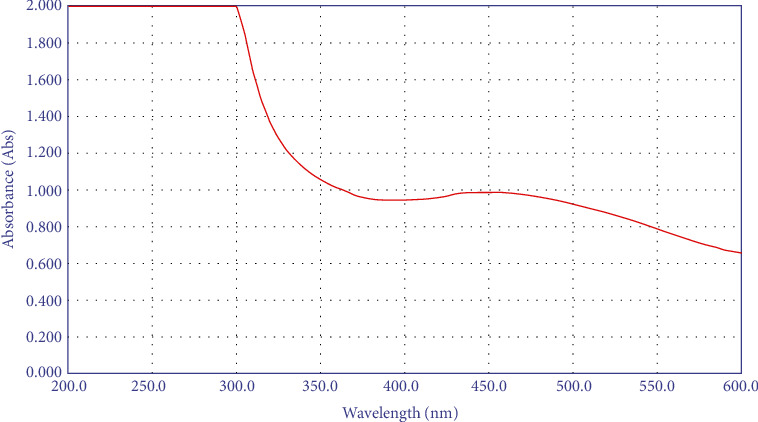
The silver surface plasmon resonance band as seen under UV-visible spectra of the bacterial filtrate.

**Figure 9:**
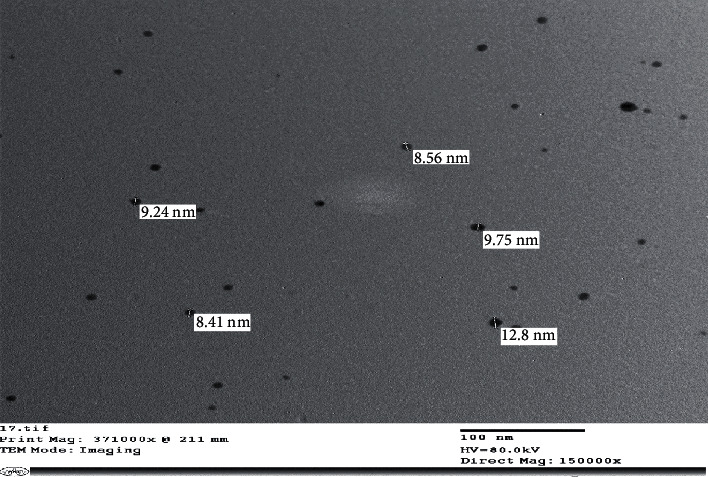
AgNPs obtained from *S*. *aureus* seen by TEM.

**Figure 10:**
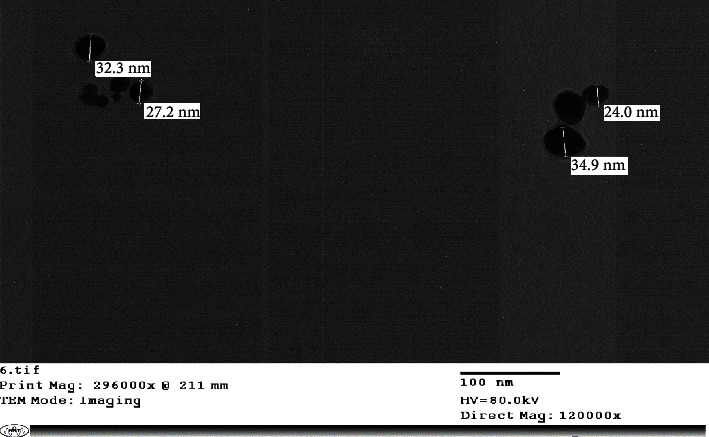
AgNPs obtained from rosemary leaves observed by TEM.

The production of reduced AgNPs in the solution was visualized under UV-vis spectrum analysis. A UV-vis spectrum is one of the most important ways of determining the formation of metal nanoparticles if the metal exhibits surface plasmon resonance. The silver surface plasmon resonance band occurs at 450 nm, as seen in Figure 8. Transmission electron microscope (TEM) images of silver nanoparticles generated from *S*. *aureus* and rosemary leaves are presented in Figures 9 and 10, respectively. AgNPs generated with *S*. *aureus* had particle sizes ranging from 8.41 to 12.8 nm, while AgNPs synthesized using *rosemary leaves* have particle sizes ranging from 24 to 34.9 nm.

## 4. Discussion

Recently, treatment of *S*. *aureus* infections using several antibiotics was less effective that led to treatment failure. Regarding this issue, VRSA and VISA strains were highly prominent [4]. Isolates of vancomycin-resistant *S*. *aureus* have emerged in many parts of the world such as Japan [15], the United States [16], and India [17]. In this study, 95 out of 100 *S*. *aureus* isolates were MRSA, and the resistance patterns of *S*. *aureus* to different antibiotics were as follows: 91% resistance for penicillin, followed by 87% for gentamicin; 85% for erythromycin; 81% for azithromycin, cefoxitin, and cefpodoxime; 75% for ceftriaxone; 69% for ciprofloxacin; 65% for clarithromycin; 62% for teicoplanin; 60% for clindamycin; 59% for levofloxacin; and 52% for doxycycline. In contrast, the sensitivity to linezolid was 80%. The agar dilution method was used for the determination of MICs for vancomycin and linezolid; the MICs were 14% for VRSA, 10% for VISA, and 11% for LRSA. Similar results were reported by Ghahremani et al. [18], who reported that resistance to penicillin was 98.8% of *S*. *aureus* isolates, followed by 61.6% for erythromycin, 50.8% for ciprofloxacin, 50.8% for clindamycin, and 17.5% for linezolid while they reported that gentamicin resistance was 41.8% and 14.1% for teicoplanin, whereas MIC results for vancomycin were 12.6% VISA and 10.5% for VRSA.

On the other hand, MRSA isolates from pediatric patients were resistant to vancomycin [19–21]. In addition, a study in Addis Ababa stated that vancomycin-resistant *S*. *aureus* isolates were only 5.1% [20]. Also, Abu Shady et al. [7] found that 4.5% of *S*. *aureus* isolates were resistant to vancomycin. The *vanA* and *vanR* genes were detected in VRSA stains while *vanY* and *vanH* were not detected in any strain. All studied genes were not detected in any VISA strain. Similar results were reported in Iran by Dezfulian et al. [10] by recording a positive *vanA* and *vanR* isolate from diabetic foot ulcer cases. Another Iranian study by Ziasistani et al. [22] reported that two VRSA strains had the *vanA*-positive gene. In addition, Ghahremani et al. [18] found that eight out of ten VRSA and one VISA isolate carried the *vanA* gene. Al-Bdery et al. [23] indicated that two out of five (40%) of VRSA were positive for the *vanA* gene. In contrast, the *vanA* gene was detected in a large number of isolates in Azhar et al.'s [24] study who reported that the *vanA* gene was detected in 74% of VRSA isolates and in Abu Shady et al. [7] who found that five out of ten (50%) VRSA were positive to *vanA*.

The silver nanoparticles were synthesized using *S*. *aureus* and *Rosmarinus officinalis*. The production of colloidal AgNPs was confirmed by the rapid appearance of a yellowish-brown color in the reaction mixture as a result of surface plasmon resonance. After 24 hours of incubation, the color observed by ocular inspection intensified, turning brown, indicating that the reaction between leaf extract or *S*. *aureus* and AgNO_3_ was completed. Similar color changes have been observed in previous studies, indicating that the interaction between leaf extract and AgNO_3_ is complete [24–26]. Characterization of AgNPs was tested by TEM, UV-vis spectroscopy, and DLS. Sizes of AgNPs differ from one study to another [25, 26] while the plasmon resonance peak range was nearly similar [26–28].

In addition, AgNPs synthesized from *S*. *aureus* and rosemary leaves had a significant antimicrobial effect with a significantly better effect of AgNPs synthesized using rosemary leaves than those synthesized using *S*. *aureus*. Nanda and Saravanan [6] evaluated the antibacterial efficacy of AgNPs produced from *S*. *aureus* against MRSA isolates and got clean zones around the wells. AgNPs can also be utilized to suppress *S*. *aureus* isolates with MICs of 5 g/ml, according to Li et al. [29]. In India, Dong et al. [26] discovered that using rosemary to make AgNPs was effective in inhibiting *S*. *aureus*. Akl et al. [11] found that silver nanoparticles generated from rosemary leaves were able to form inhibition zones against *S*. *aureus* utilizing the disc diffusion method.

According to our study, there was a significant increase in the vancomycin effect on VRSA strains when added to AgNPs. Previous studies have also reported this synergistic effect [30, 31]. This can result in decreasing vancomycin resistance among *S*. *aureus* isolates. MICs obtained from pure vancomycin were significantly decreased after capping with AgNPs [27, 28]. The mean zones of inhibition were increased when vancomycin was added to AgNPs compared to vancomycin alone [32].

Rosemary extracts have been used in the treatment of diseases and in food preservation because they prevent oxidation and microbial contamination. *Rosmarinus officinalis* is a rich source of phenolic compounds and terpenoids, as well as reducing sugar, flavonoids, and alkaloids, and their properties are derived from its extracts which are used for the treatment of illnesses and in food preservation due to its antimicrobial effect on bacteria responsible for food spoilage such as *S*. *aureus*. These compounds give a synergistic effect with silver nitrate when AgNPs were synthesized [33].

## 5. Conclusion

In conclusion, VRSA isolates came from a wide range of genetic backgrounds, which should be properly considered. The resistance to vancomycin, the main treatment against MRSA and MSSA, has been unpredictably high in this study. To avert the scenario of untreatable *S*. *aureus*, novel medications or strategies to improve current alternatives are needed. Silver nanoparticles (AgNPs) are a new class of antimicrobials that can help kill *S*. *aureus* isolates when other antibiotics have failed, such as vancomycin. To achieve satisfactory results *in vivo*, more research is needed.

## Figures and Tables

**Table 1:** Nucleotide sequences of PCR primers for vanA cluster genes.

Primer pair	Nucleotide sequences	Annealing temperature	Expected product size (bp)
vanR F vanR R	AGCGATAAAATACTTATTGTGGA CGGATTATCAATGGTGTCGTT	62°C	645
vanH F vanH R	ATCGGCATTACTGTTTATGGAT TCCTTTCAAAATCCAAACAGTTT	60°C	943
vanA F vanA R	ATGAATAGAATAAAAGTTGCAATAC CCCCTTTAACGCTAATACGAT	62°C	1029
vanY F vanY R	ATGAAGAAGTTGTTTTTTTTA TTACCTCCTTGAATTAGTAT	55°C	912

**Table:** (a) The antimicrobial effect of AgNPs synthesized using *S*. *aureus*

Sample no.	Mean ZOI ± SE for AgNPs (mm)	ZOI ± SE for VAN disc (mm)	*P* value for VAN and ZOI AgNPs	ZOI ± SE for VAN+AgNPs (mm)	*P* value for ZOI VAN and VAN+AgNPs
1	18 ± 0.408	14 ± 0.741	*P* ≤ 0.05	21 ± 1.149	*P* ≤ 0.05
2	17 ± 0.421	15 ± 0.652		27 ± 1.009	
3	15 ± 0.448	10 ± 1.144		18 ± 0.978	
4	15 ± 0.577	11 ± 1.087		16 ± 1.243	

ZOI: zone of inhibition; VAN: vancomycin; SEM: standard error mean.

**Table:** (b) The antimicrobial effect of AgNPs synthesized using rosemary leaves

Sample no.	Mean ZOI ± SE for AgNPs (mm)	ZOI ± SE for VAN disc (mm)	*P* value for VAN and ZOI AgNPs	ZOI ± SE for VAN+AgNPs (mm)	*P* value for ZOI VAN and VAN+AgNPs
1	24 ± 1.168	14 ± 0.741	*P* ≤ 0.05	23 ± 1.354	*P* ≤ 0.05
2	20 ± 1.379	15 ± 0.652		24 ± 1.255	
3	21 ± 0.988	10 ± 1.144		20 ± 1.195	
4	19 ± 1.249	11 ± 1.087		19 ± 1.177	

ZOI: zone of inhibition; VAN: vancomycin; SEM: standard error mean.

**Table 3:** Mean and standard error for all zones of inhibition for AgNPs and vancomycin.

Mean zone of inhibitions (*n* = 4)	Mean ± SE
AgNPs from *S*. *aureus*	16.25 ± 0.750
AgNPs from rosemary leaves	21.00 ± 1.080
Vancomycin disc	12.50 ± 1.190
Vancomycin+AgNPs from *S*. *aureus*	20.50 ± 2.398
Vancomycin+AgNPs from rosemary leaves	21.50 ± 190

**Table:** (a) Stability of silver nanoparticles synthesized from *S*. *aureus* after 24 hours, 1 month, 3 months, and 6 months

Sample no.	ZOI ± SE after 1 day (mm)	ZOI ± SE after 1 month (mm)	ZOI ± SE after 3 months (mm)	ZOI ± SE after 6 months (mm)	*P* value
1	18 ± 0.408	17 ± 0.677	18 ± 0.876	17 ± 0.933	*P* > 0.05
2	17 ± 0.421	18 ± 0.645	17 ± 0.763	17 ± 1.122	
3	15 ± 0.448	16 ± 0.799	14 ± 0.655	14 ± 1.242	
4	15 ± 0.577	15 ± 0.597	16 ± 0.757	15 ± 1.154	

**Table:** (b) Stability of silver nanoparticles synthesized from rosemary leaves after 24 hours, 1 month, 3 months, and 6 months

Sample no.	ZOI ± SE after 1 day (mm)	ZOI ± SE after 1 month (mm)	ZOI ± SE after 3 months (mm)	ZOI ± SE after 6 months (mm)	*P* value
1	24 ± 1.168	22 ± 1.233	23 ± 1.431	23 ± 1.249	*P* > 0.05
2	20 ± 1.379	21 ± 1.354	20 ± 1.376	20 ± 1.133	
3	21 ± 0.988	20 ± 1.187	20 ± 1.345	19 ± 1.379	
4	19 ± 1.249	19 ± 1.089	18 ± 1.234	18 ± 1.174	

ZOI: zone of inhibition; VAN: vancomycin.

## Data Availability

Data are available upon request through the corresponding author Prof. Haddad El Rabey (elrabey@hotmail.com).

## Abbreviations

AgNPs: Silver nanoparticles
CLSI: Clinical and Laboratory Standards Institute
DLS: Dynamic light scattering
LRSA: Linezolid-resistant *Staphylococcus aureus*
*M*: Mean value
MIC: Minimum inhibitory concentration
MRSA: Methicillin-resistant *Staphylococcus aureus*
PTA: Phosphotungtic acid
SE: Standard error
TEM: Transmission electron microscope
TEM: Transmission electron microscopy
UV-vis: Ultraviolet-visible
VRSA: Vancomycin-resistant *Staphylococcus aureus*
ZOI: Zone of inhibition.

## Supplementary Materials

Supplementary Materials Supplementary Figure 1: the color of the solution changed to yellowish-brown after *S*. *aureus* was added to AgNO_3_, indicating the production of AgNPs.
