# Supplementary material for: Inhibition of the Vancomycin Resistance in Staphylococcus aureus in Egypt Using Silver Nanoparticles
Source: Biomed Res Int. 2022 Apr 30;2022:7380147. doi: 10.1155/2022/7380147 (PMC9078785; doi:10.1155/2022/7380147)
Supplement: Supplementary Materials — Supplementary Figure 1: the color of the solution changed to yellowish-brown after S. aureus was added to AgNO3, indicating the production of AgNPs. [file 7380147.f1.docx]

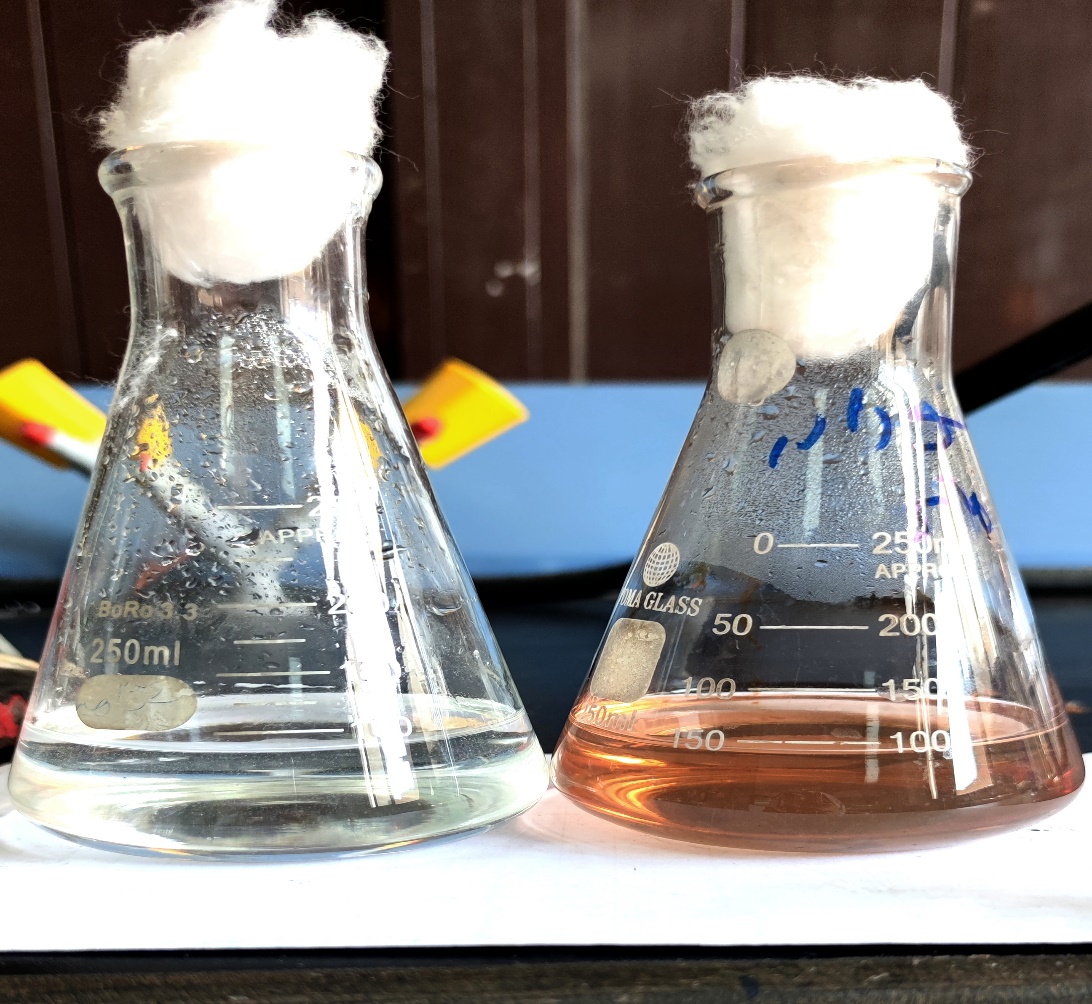


Supplementary Figure (1): The color of the solution changed to yellowish brown after *S. aureus* was added to AgNO3, indicating the production of AgNPs.
